# Analysis of Milk Using a Portable Potentiometric Electronic Tongue Based on Five Polymeric Membrane Sensors

**DOI:** 10.3389/fchem.2021.706460

**Published:** 2021-07-05

**Authors:** C. Pérez-González, C. Salvo-Comino, F. Martin-Pedrosa, L. Dias, M. A. Rodriguez-Perez, C. Garcia-Cabezon, M. L. Rodriguez-Mendez

**Affiliations:** ^1^ Group UVASENS, Escuela de Ingenierías Industriales, Universidad de Valladolid, Valladolid, Spain; ^2^ BioecoUVA Research Institute, Universidad de Valladolid, Valladolid, Spain; ^3^ Dpt. of Materials Science, Universidad de Valladolid, Valladolid, Spain; ^4^ Centro de Investigação de Montanha (CIMO), ESA, Instituto Politécnico de Bragança, Bragança, Portugal

**Keywords:** potentiometric electronic tongue, milk, dairy, fat content, lactose, acidity

## Abstract

A portable potentiometric electronic tongue (PE-tongue) was developed and applied to evaluate the quality of milk with different fat content (skimmed, semi-skimmed, and whole) and with different nutritional content (classic, calcium-enriched, lactose-free, folic acid–enriched, and enriched in sterols of vegetal origin). The system consisted of a simplified array of five sensors based on PVC membranes, coupled to a data logger. The five sensors were selected from a larger set of 20 sensors by applying the genetic algorithm (GA) to the responses to compounds usually found in milk including salts (KCl, CaCl_2_, and NaCl), sugars (lactose, glucose, and galactose), and organic acids (citric acid and lactic acid). Principal component analysis (PCA) and support vector machine (SVM) results indicated that the PE-tongue consisting of a five-electrode array could successfully discriminate and classify milk samples according to their nutritional content. The PE-tongue provided similar discrimination capability to that of a more complex system formed by a 20-sensor array. SVM regression models were used to predict the physicochemical parameters classically used in milk quality control (acidity, density, %proteins, %lactose, and %fat). The prediction results were excellent and similar to those obtained with a much more complex array consisting of 20 sensors. Moreover, the SVM method confirmed that spoilage of unsealed milk could be correctly identified with the simplified system and the increase in acidity could be accurately predicted. The results obtained demonstrate the possibility of using the simplified PE-tongue to predict milk quality and provide information on the chemical composition of milk using a simple and portable system.

## Introduction

Milk is a nutritious food containing significant components including fats, lactose, sugars, amino acids, vitamins, nucleotides, inorganic salts, and trace elements among many others. Milk composition and nutrient levels are usually assessed by classic analytical techniques such as gas chromatography/mass spectroscopy, high-performance liquid chromatography, and spectroscopy (Toldrá et al., 2021). However, there is a great interest in developing new methods for rapid detection, quantification, and evaluation of milk. Electrochemical sensors can be an alternative to classical laboratory techniques because of their high sensitivity, low cost, and inherent portability (Zeng et al., 2018). However, electrochemical sensors have a lack of selectivity, and this can be a problem when dealing with complex samples such as milk.

An interesting approach to improve the performance of non-specific sensors consists in the use of arrays of sensors. According to the IUPAC definition, an electronic tongue (ET) is a multisensor system, which consists of a number of low-selective sensors and uses advanced mathematical procedures for signal processing based on pattern recognition and/or multivariate data analysis (Vlasov et al., 2005; Rodríguez-Méndez, 2016). Several types of sensors have been used in ETs dedicated to the analysis of milk and dairy products (Poghossian et al., 2019). These include voltammetric electrodes (Cetó et al., 2014; Wei et al., 2013; Bougrini et al., 2014; Salvo-Comino et al., 2018) or impedimetric sensors (Scagiona et al., 2016). However, most of the works in ETs applied to milk have been carried out using potentiometric sensor arrays. Since the pioneering work of Toko’s group that developed an ET based on potentiometric electrodes (composed of several lipid/polymer membranes) (Hayaschi et al., 1995), the analysis of milk samples using ETs has been an active field of research. These instruments have been constantly progressing, and new improvements in sensor arrays, in data processing methods, and in applications are reported in the literature (Ciosek and Wroblewski, 2011; Ciosek and Wroblewski, 2015).

Potentiometric ETs have been used to analyze different aspects related to the quality of milk and dairy products. For example, they have been used for the discrimination and classification of milk based on fat content and brand (Ciosek and Wroblewski, 2008); for the classification of natural, fermented, and UHT milk (Tazi et al., 2018); to evaluate the organoleptic properties (Minghui et al., 2019); to detect flavor changes in bovine and goat milk (Tazi et al., 2017); in the detection of spoilage (Poghossian et al., 2019); and in the detection of adulterations of goat milk with bovine milk (Dias et al., 2009) or detection of water added to dairy products (Mabrook et al., 2006) among others. Only a few works have explored the possibility of using e-tongues to evaluate chemical parameters in milk. These works use partial least squares (PLS) models to establish correlations between the signals obtained with the sensor arrays and the chemical parameters obtained by classical techniques. For example, e-tongues have been used to determine the content of ethanol, acetaldehyde, lactic acid, acetic acid, and citric acid in probiotic fermented milk (Hruskar et al., 2010), or for the simultaneous detection of water-soluble ions (Torabi et al., 2020).

In many cases, potentiometric e-tongues consist of a large number of sensors (typically between 8 and 36) (Dias et al., 2009; Podrażka et al., 2018; Torabi et al., 2020). The idea behind these large arrays is that a higher number of sensing units with different selectivity and sensitivity can provide a larger amount of information, improving the discrimination and prediction capability of the e-tongue. However, a large number of sensors imply a considerable number of variables to manage, and in many cases on-line. In addition, a large number of variables imply the presence of features containing irrelevant or redundant information. In addition, collinearity present in the variables may affect the prediction results (Leardi, 1998). Finally, when models are built using a large number of variables, the training sample set must also be large. All these reasons make it interesting to reduce the number of variables by selecting only those that provide important information.

Several methods have been used to reduce the number of variables in e-tongues, including the wavelet transform (Holmin et al., 2002) or the kernel method (Salvo-Comino et al., 2018). Tools such as a genetic algorithm (GA) applied to PLS regression can be successfully used as a feature selection technique in voltammetric e-tongues (Prieto et al., 2013). The GA represents an efficient approach to non-linear optimization problems and has several advantages. For example, it does not require linear assumptions and is independent of the misfit criterion (Mirjalili, 2019; Reeves, 2010). The GA incorporates and exploits data collected during model space sampling, resulting in an incredibly efficient and robust optimization technique (Beg and Islam, 2016).

The main goal of this study was to develop a simplified and portable potentiometric electronic tongue (PE-tongue). To this end, the work has been carried out to 1) reduce the number of sensors forming the array using the GA; 2) investigate whether this simplified system could be used to discriminate milk with different fat content and nutritional composition using principal component analysis (PCA); 3) investigate whether acidity, density, %proteins, %lactose, and %fat could be accurately predicted using support vector machine (SVM) regression; and 4) evaluate the ability of the PE-tongue to detect spoilage of unsealed milk while exploring the possibility of predicting changes in physicochemical properties.

## Materials and Methods

All the reagents used in this work were of analytical degree and used as supplied. Standard solutions of KCl, CaCl_2_, NaCl, lactose, glucose, galactose, citric acid, and lactic acid (Sigma-Aldrich, St. Louis, United States) were prepared in MilliQ deionized water (Merck KGaA, Darmstadt, Germany). Two sets of milk samples were used in the study. The first set was used to train the e-tongue and consisted of 13 types of milk samples (five replicas of each, total of 65 samples), including milk with different fat content (skimmed, semi-skimmed, and whole) and milk with different nutritional content (classic, calcium-enriched, lactose-free, folic acid–enriched, and enriched in sterols of vegetal origin) (Table 1). A second set of five samples was used as an external testing set to validate the results obtained with the e-tongue. The samples were stored at room temperature until used. Milk samples were analyzed by classical chemical methods: acidity (titration method ISO 22113:2012), density (hydrometer method ISO 2449:1974), fat (Röse-Gottlieb gravimetric method ISO 1211:2010), proteins (Kjeldahl method ISO 8968–1:2014), and lactose content (HPLC ISO 22662:2007). The averages of physicochemical parameters analyzed for each of the milk samples are collected in Table 1.

**TABLE 1:** Types of milk samples included in the study and results of the chemical analysis.

Sample		Acidity (ºD)	Density (g/ml)	Fat (%m)	Proteins (%m)	Lactose (%m)
Training set						
C-S	Classic—skimmed	12.65	1033.55	0.32	3.3	5
C-SS	Classic—semi-skimmed	12.55	1031.6	1.56	3.27	4.91
C-W	Classic—whole	12.17	1029.38	3.56	3.21	4.85
CA-S	Calcium—skimmed	15.82	1039.47	0.29	3.93	5.59
CA-SS	Calcium—semi-skimmed	16.06	1037.29	1.55	3.9	5.49
CA-W	Calcium—whole	15.86	1035.71	3.55	3.91	5.54
L-S	Lactose-free—skimmed	12.67	1033.57	0.31	3.29	0.36
L-SS	Lactose-free—semi-skimmed	12.19	1032.09	1.59	3.31	0.42
L-W	Lactose-free—whole	11.98	1029.4	3.59	3.23	0.31
F-S	Folic acid—skimmed	12.57	1033.7	0.40	3.29	4.95
F-SS	Folic acid—semi-skimmed	12.95	1032.38	1.64	3.21	4.93
F-W	Folic acid—whole	12.72	1030.55	3.1	3.18	4.94
P-SS	Proactive—semi-skimmed	12.28	1031.48	1.91	3.16	4.88
External validation set						
V1	Skimmed calcium	15.94	1,039	0.35	3.89	5.49
V2	Semi-skimmed classic	12.36	1,032.14	1.58	3.34	4.97
V3	Semi-skimmed classic	12.45	1,032.45	1.65	3.35	4.96
V4	Whole acid folic	12.95	1,030.92	3.12	3.29	4.89
V5	Whole free lactose	12.45	1,029.92	3.58	3.33	0.52

Sensors were based on polymeric membranes using high-density polyvinyl chloride (PVC) as the polymeric matrix (Sigma-Aldrich, St. Louis, United States). Additives and plasticizers were added to the polymeric matrix using tetrahydrofurane as the solvent (Sigma-Aldrich, St. Louis, United States). The final composition was 32% of PVC, 65% of the plasticizer compound, and 3% of the additive.

The additives and plasticizers are listed in Table 2. Five plasticizers (A–F) were combined with four additives (1–4) to obtain 20 different sensors that were named A1, A2, A3, A4; B1, B2, B3, B4; C1, C2 C3, C4; D1, D2, D3, D4; and F1, F2, F3, F4.

**TABLE 2:** List of additives and plasticizers.

Component		Nomenclature
Additives	Octadecylamine	1
	Oleyl alcohol	2
	Tridodecylmethylammonium chloride	3
	Oleic acid	4
Plasticizers	Bis(1-butylpentyl)adipate	A
	Tris(2-ethylhexyl)phosphate	B
	Dibutyl sebacate	C
	2-Nitrophenyl-octylether	D
	Dioctyl phenylphosphonate	F

**FIGURE 1:**
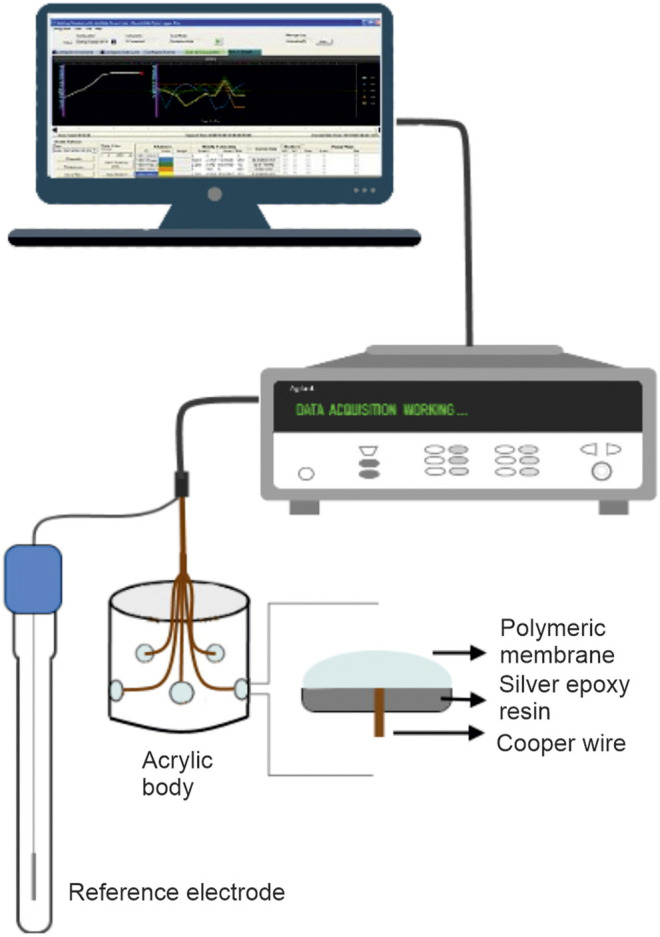
Scheme of the potentiometric e-tongue used in this work.

The body of the e-tongue consisted of an acrylic tube in which 20 holes of 0.3 cm diameter were drilled. Each hole was filled with a conductive silver epoxy resin (EPO-TEK, Billerica, United States) and connected to a multiplexer (Agilent Data Acquisition Switch Unit 34970A) *via* electrical copper wires. The outer surface of each hole was covered with one of the polymeric membranes described in Table 2. An Ag/AgCl electrode was used as the reference electrode for all measurements. Figure 1 shows the schematic of the e-tongue system containing the working and reference electrodes connected to a multiplexer.

The sensor array was immersed in a glass cell containing 100 ml of each sample (diluted 1:1 in deionized water). Potentiometric measurements were recorded for 5 min by registering the sensor signals every 3 s. All the samples were measured in quintuplicate. Throughout the experiment, the samples were kept at room temperature and under gentle agitation.

The statistical analysis was performed using RKWard 0.7.1 and Matlab R2014b (The Mathworks Inc., Natick, United States). Data analysis included pre-processing of the potentiometric signals using the genetic algorithm and partial least squares (GA–PLS) procedure. Principal component analysis was used to assess the discrimination ability of the multisensor system. Support vector machine regression (SVMR) was used to establish correlations between the results obtained with the e-tongue and the chemical parameters given by chemical analysis. In addition, SVMR was used as a classification method to predict the quality of milk samples.

## Results

### Characterization of the Individual Sensors

The performance of the 20 PVC membrane–based potentiometric sensors was evaluated using eight standard solutions of compounds commonly present in milk, including salts (KCl, CaCl_2_, and NaCl), sugars (lactose, glucose, and galactose), and organic acids (citric acid and lactic acid), with concentrations ranging from 1 × 10^–4^ to 1 × 10^–1^ mol/L.

**FIGURE 2:**
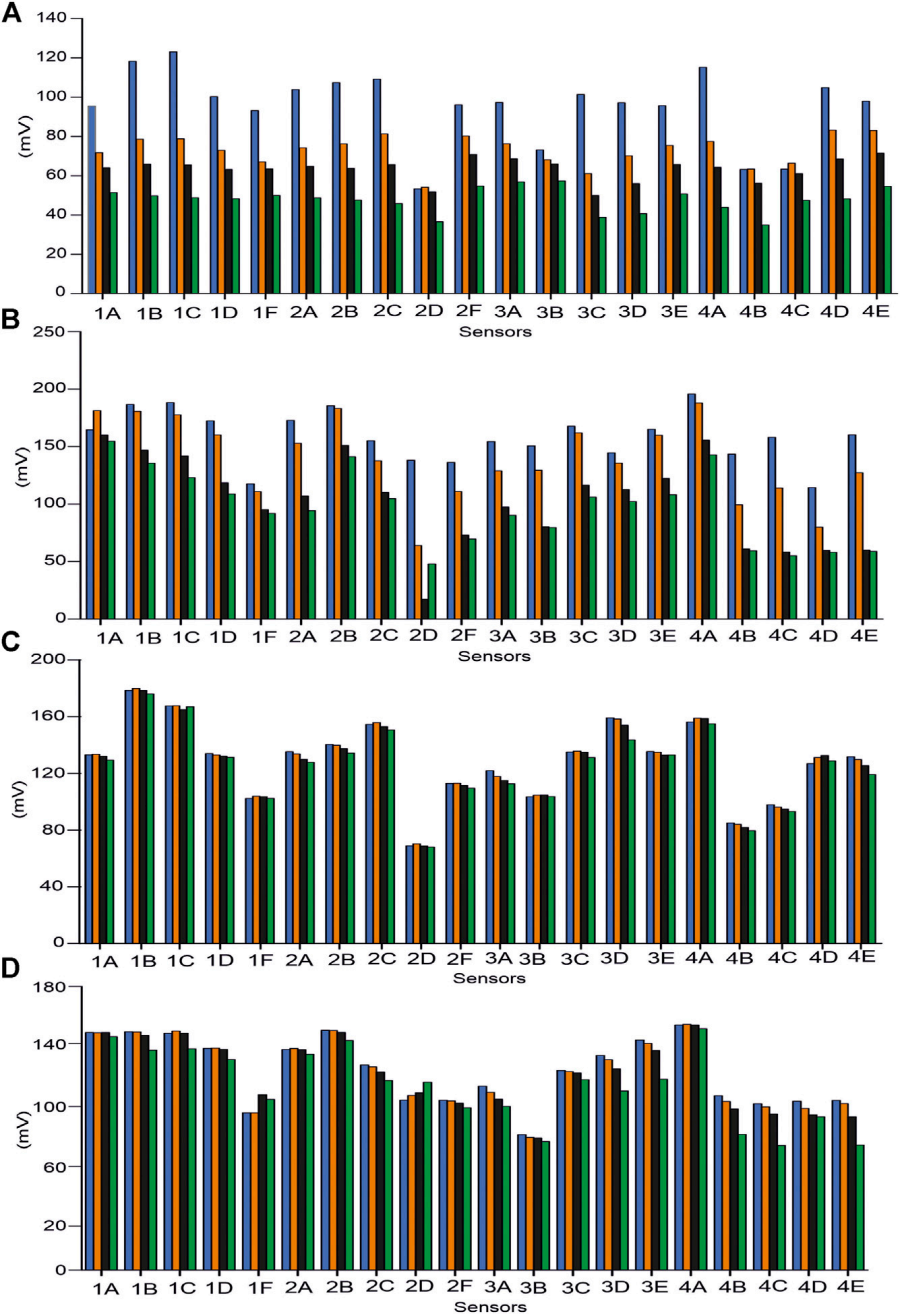
Responses of the array of sensors to standard solutions of **(A)** CaCl_2_, **(B)** lactic acid, **(C)** lactose, and **(D)** galactose. Measures were carried out at four concentrations: 5 × 10^–1^ M (blue), 2 × 10^–1^ M (orange), 1 × 10^–1^ M (black), and 10^–2^ M (green) for ionic salts and 10^–1^ M (blue), 10^–2^ M (orange), 10^–3^ M (black), and 10^–4^ M (green) for lactic acid, lactose, and galactose.

After immersing the electrodes in the corresponding solution, membrane potentials were recorded for 5 min every 3 s until stabilization of the signals. Signals were considered stable when an average variation of 1.6 mV/decade was observed between each reading. Figure 2 illustrates the responses obtained when the sensors were immersed in standard solutions (figure shows the average of five replicas). This figure shows an example of salt (CaCl_2_) and acid (lactic acid) of monosaccharide (galactose) and of disaccharide (lactose). The figure also includes the responses obtained at four different concentrations (5 × 10^−1^ M, 2 × 10^−1^ M, 1 × 10^−1^ M, and 10^–2^ M) for ionic salts and 10^−1^ M, 10^–2^ M, 10^–3^ M, and 10^–4^ M for the rest of the compounds. As shown in Figure 2, the sensors showed a variety of responses to the different components of milk. The sensitivity values shown in Table 3 (measured as the slope of the calibration curves) were lower for sugars than for solutions containing ions and lactic acid, confirming the different reactivity of the sensors to components usually found in milk matrices. These differences confirm the cross-selectivity of the sensors and their suitability to be part of a multisensor system.

**TABLE 3:** Sensitivity values obtained from the slopes of the calibration curves.

	NaCl	CaCl_2_	KCl	Lactose	Glucose	Galactose	Citric acid	Lactic acid
1A	81.30	78.37	58.06	16.13	18.62	10.23	35.98	46.24
1B	105.97	140.62	106.57	6.34	53.33	47.18	71.89	82.15
1C	133.63	128.83	118.81	15.08	58.38	47.18	38.40	88.65
1D	55.27	93.92	57.80	17.86	45.32	30.83	86.29	86.35
1F	75.25	64.44	32.41	−3.58	42.74	23.18	91.10	38.55
2A	86.73	98.96	81.74	46.61	62.42	−6.53	106.15	117.24
2B	69.79	109.17	76.80	27.60	35.56	15.69	100.90	75.27
2C	76.48	119.76	83.69	3.55	24.73	31.23	90.14	67.26
2D	80.30	37.77	70.35	0.37	26.92	56.74	114.94	123.56
2F	27.36	78.08	50.58	19.43	33.69	−12.14	85.45	86.45
3A	28.40	73.52	52.15	70.82	31.30	26.24	74.16	80.35
3B	18.37	29.75	15.46	8.06	87.40	72.91	96.37	115.37
3C	71.06	105.96	93.75	21.43	17.40	18.99	82.57	111.33
3D	65.93	109.58	86.87	76.46	86.20	81.04	36.42	64.25
3E	60.02	87.03	60.11	9.20	68.40	81.96	105.12	79.21
4A	102.35	127.67	105.21	−6.23	8.82	8.88	68.86	79.66
4B	65.80	63.98	24.07	40.01	39.74	78.39	101.56	83.27
4C	34.01	41.90	35.01	33.15	79.34	59.42	100.26	122.36
4D	113.37	105.73	64.77	−4.68	72.40	85.15	75.62	45.62
4E	34.85	86.26	57.28	79.23	84.50	80.32	97.65	126.32

**FIGURE 3:**
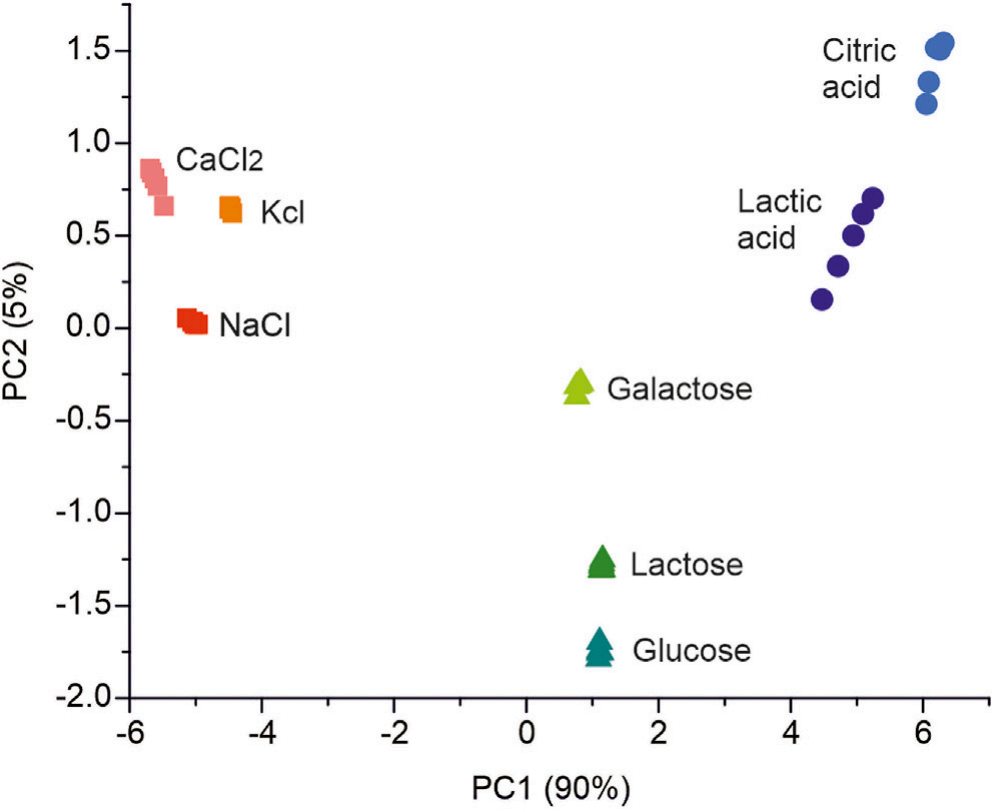
PCA score plot of the standard solutions of compounds present in milk.

The signal repeatability and the reproducibility of the sensors against standard solutions were analyzed. Repeatability was evaluated by analyzing the responses of the sensors immersed in 0.1 M KCl solutions. Measurements were performed in quintuplicate, and coefficients of variation between 0.1 and 1.38% were obtained for all sensors. Reproducibility was calculated by analyzing the responses of two sets of identical sensors immersed in 0.1 M KCl solution. The responses of the sensors showed coefficients of variation between 0.57 and 7.76%. The lifetime was studied by calculating the coefficients of variation of the responses of the sensors immersed in 0.1 M KCl solution for a period of thirty days. The results showed coefficients of variation between 0.52 and 8.56%. The data collected with the set of 20 sensors were used as input variables for multivariate analysis. PCA was used to assess the discrimination ability of the array. Figure 3 shows the score plot of this analysis, in which the first two principal components explained 95% of the covariance of the data (90% by PC1 and 5% by PC2). The compounds analyzed were grouped according to their chemical nature. Ionic compounds appear on the left side of the graph, sugars in the middle, and organic acids in the right part of the diagram.

### Analysis of Milk: Simplification of the Array of Sensors

**FIGURE 4:**
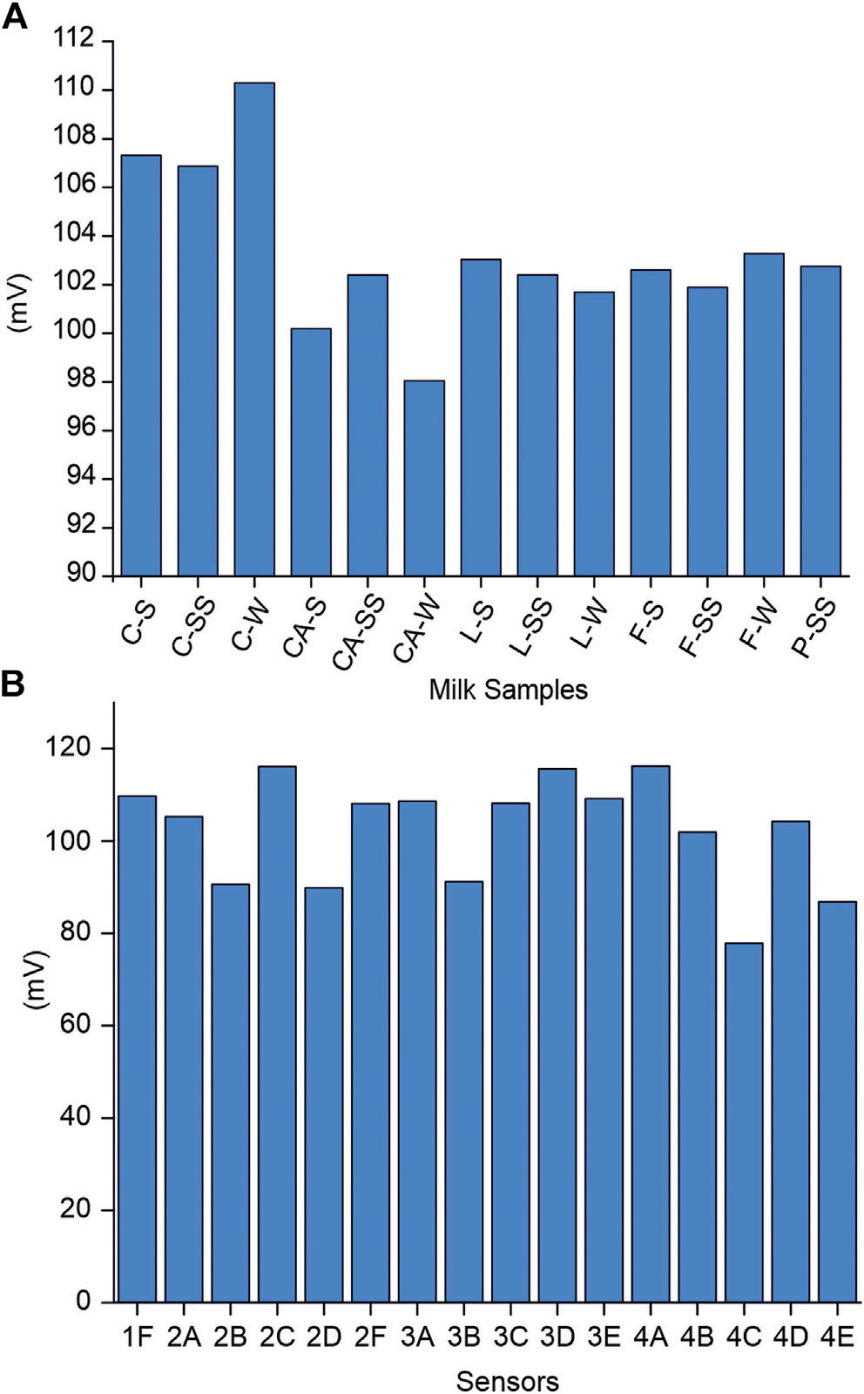
**(A)** Potentiometric responses of sensor 2C toward milk. **(B)** Response of the array of 20 sensors to classic whole milk (C-W).

The performance capabilities of the 20-sensor array were evaluated by registering the potentiometric signals obtained when the electrodes were immersed in milk samples of different qualities. Figure 4 illustrates the responses of the sensors to the milk samples analyzed. As can be seen, each sensor shows distinct responses toward milk with different composition. For example, classic milk showed higher potentials than calcium-enriched milk or lactose-free milk, regardless of their fat content. Moreover, all sensors showed significant differences between samples, confirming the cross-selectivity of the array.

The e-tongue developed here must operate in an industrial environment where time and cost are of paramount importance. For this reason, it is relevant to simplify the system by reducing the number of sensors included in the array but without losing information. A visual inspection of the sensor array responses indicated that some sensors could provide redundant information. For example, the responses of sensor 4A to different sugars were quite similar, and sensor 1F could barely detect sugars. To reduce the number of sensors in the array, the GA procedure was chosen to select the best sensors to be included in the array and build predictive regression models (Gendreau and Potvin, 2010). With GA, it is possible to get good solutions for the optimization problems. The GA was applied separately for the five chemical parameters (acidity, density, proteins, lactose, and fat). Fitting a PLS model (GA–PLS) to the sensor array for 13 milk samples and computing the performance by a leave-one-out cross-validation procedure was the way to optimize the problem. The chosen probability of initial variable selection was 0.5, the probability of crossover was 0.5, and the probability of mutation was 0.1. The selected variables were determined to be optimal after 500 GA–PLS evaluations with changing empirical parameter values. Ten iterations per evaluation were performed to avoid overfitting.

**FIGURE 5:**
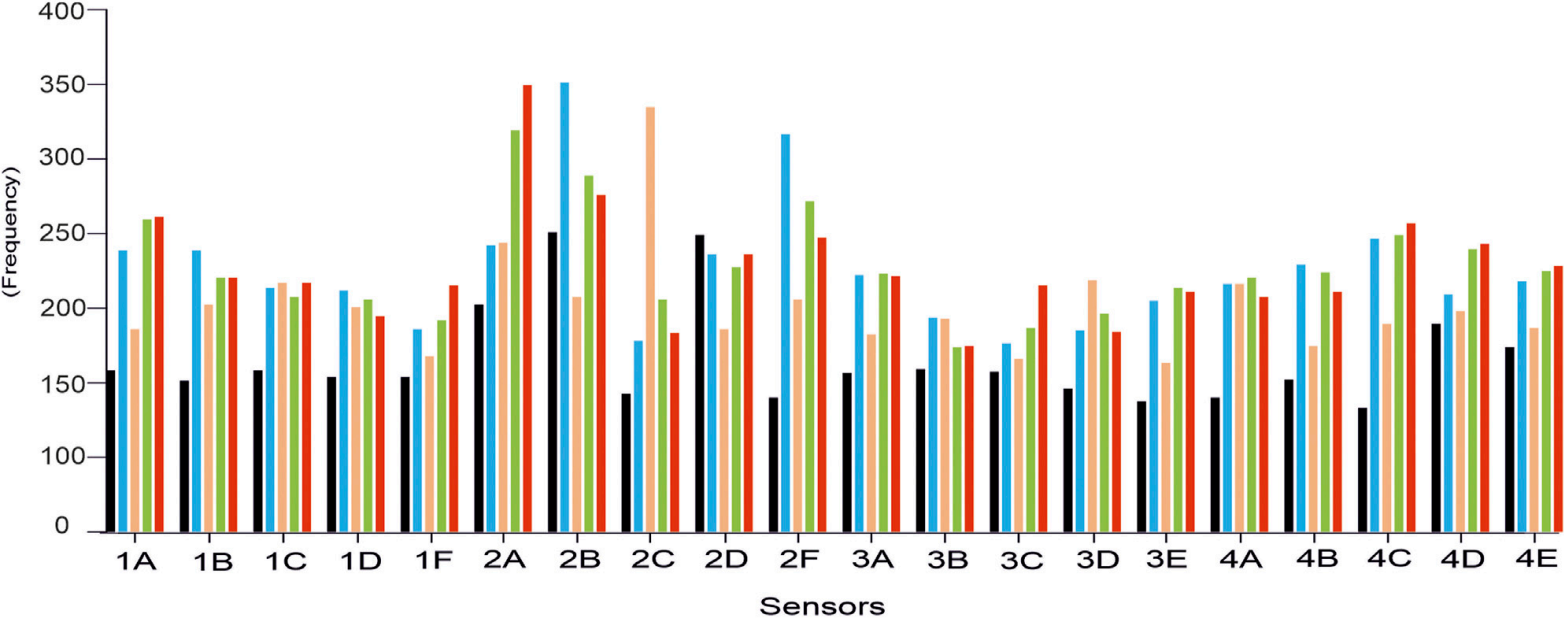
Frequency of appearance of the sensors for 500 GA–PLS evaluations, for each of the empirical parameters: density (black), acidity (blue), fat (orange), protein (green), and lactose content (red).

The response variable for each GA–PLS optimization was a vector of zeros and ones, corresponding to the thirteen milk types. In this way, the GA–PLS searches for the most relevant sensors in the electronic tongue to identify differences related to milk type. The sensors that provided a greater amount of information are indicated by showing higher responses in Figure 5. These sensors provided a better differentiation between samples according to the type of milk by nutritional content.

As can be seen, the sensors that include oleyl alcohol in their composition showed the best responses for each of the parameters studied. Therefore, these sensors were selected to form a new simplified e-tongue consisting of five sensors (2A, 2B, 2C, 2D, and 2F). The discrimination and prediction capability of the simplified e-tongue was studied.

### Evaluation of the Performance of the 20-Sensor Array and the Simplified System Based on Five Sensors

1) Assessment of the discrimination capacity using PCA

In this section, the performance of the 20-sensor–based e-tongue was compared with that of the five-sensor–based system.

**FIGURE 6:**
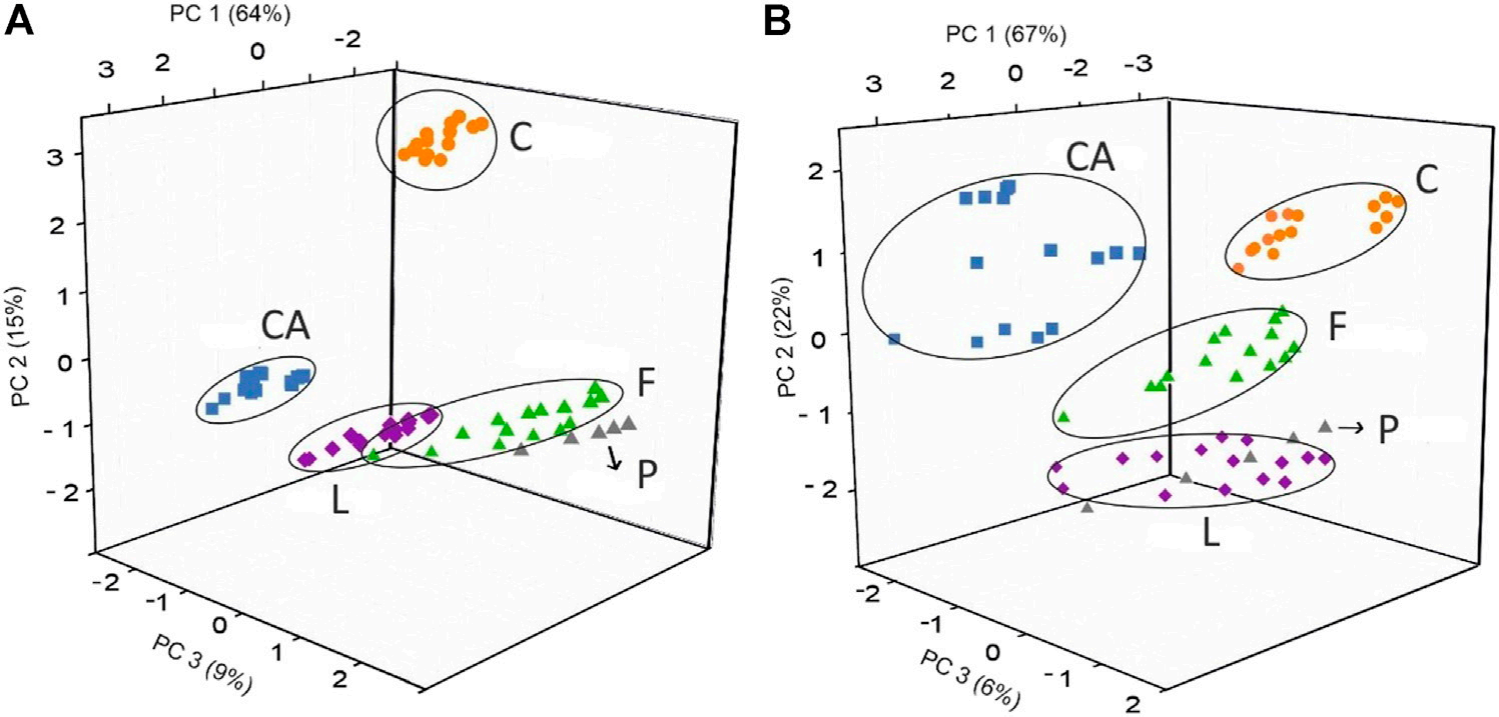
PCA score plot of the 13 milk samples of different fat content and nutritional characteristics analyzed using **(A)** a 20-sensor array and **(B)** a five-sensor array. C = classic milk, CA = calcium-enriched milk, L = lactose-free milk, F = folic acid–enriched milk, and P = vegetal sterol–enriched milk.

A PCA was performed to evaluate the discrimination ability of the array. Figure 6A shows the score plot of this analysis, in which the first three principal components jointly explained 88% of data variability (64% by PC1, 15% by PC2, and 9% by PC3). Surprisingly, discrimination between milk samples was not dominated by fat content. Instead, milk samples were grouped according to added components (calcium or folic acid) or removed components (lactose). PC1 clearly discriminated classic milk (C: includes milk samples C-S, C-SS, and C-W) regardless of their fat content from the rest of the milk samples. PC2 discriminated calcium-enriched milk (CA: includes milk samples CA-S, CA-SS, and CA-W) from lactose-free milk (L: includes milk samples L-S, L-SS, and L-W) regardless of their fat content. Milk enriched in folic acid or in vegetal sterols appeared at the bottom of the diagram (F and P: include F-S, F-SS, F-W, and P-SS). The mixing of samples among some groups may be due to the sensitivity of the sensor device to other milk components such as fat content, which widely affect physicochemical aspects such as viscosity or density in the samples.

Figure 6B shows the score plot obtained from the responses to milk with different nutritional content obtained using the PE-tongue based on a five-sensor array. The first two PCs explain 89% of the total variance. As can be seen in Figure 6B, milk samples with similar nutritional content appeared in the same region of the graph. The first group contains milk with enhanced nutritional calcium content (CA: includes CA-S, CA-SS, and CA-W); the second group includes milk samples without any modification in terms of nutritional content (C: includes C-S, C-SS, and C-W); the third group contains milk that has been modified for low lactose content (L: includes L-S, L-SS, and L-W); finally, there is a mixed group with four types of milk samples with an increased amount of folic acid in their composition (F and P: include F-S, F-SS, F-W, and P-SS). The first and second groups, in the positive part of the second principal component, are separated by the first principal component (being in the positive and negative parts, respectively). The third, fourth, and fifth groups are separated by the second and third principal components (in the negative region) with a very low contribution of the first principal component.

This result indicates that the final subset of variables retained by GA–PLS presents the ability to discriminate milk classes according to their nutritional components, similar to that obtained with a complex system consisting of 20 sensors, although a complete discrimination has not been obtained.

2) Assessment of the classification capacity using SVM

The classification capability of the PE-tongue system was tested using SVM using the radial basis function (RBF) (Wu and Wang 2009) as a non-linear kernel, defined as follows:

$$K\left(x_{i}-x_{j}\right)=\exp\left(-\gamma {\left\|x_{i}-x_{j}\right\|}^{2}\right),\gamma >0,$$

where *x*_*i*_ and *x*_*j*_ are the training vectors of the input data and *γ* is the kernel parameter.

This kernel has been chosen since the number of instances is larger than the number of features, and therefore, it is recommended to use non-linear kernels (Hsu 2016).

In this study, the SVM model was trained with data obtained from 65 samples (corresponding to the thirteen types of milk samples with different fat content and nutritional characteristics). The same scaling factors were applied for the training and testing sets.

The optimal SVM regularization parameter (C, which is the penalty parameter of the error term) was set to the highest value (C = 100), implying that classification errors were not tolerated for the set of response patterns used for SVM training. Due to the relatively small number of available measurements, the leave-one-out cross-validation method was implemented to better estimate the true success rate that could be achieved with the SVM. This assumes that, with the given n measurements, the model was trained n times using n − 1 training vectors. This vector was then used for testing.

The results obtained for the 20-sensor array showed an accuracy of 99.87% for the classification of the 13 classes in training and 98.46% in validation. The results showed that the accuracy of the five-sensor array training set was 96.92%, and those of the validation set showed an accuracy of 90.76%, for the thirteen categories. Each of the problem samples was correctly classified according to its nutritional content. These results determine that the electronic tongue developed with five sensors was able to classify the milk samples according to their nutritional content and also by their fat content.

3) Prediction of chemical parameters by means of support vector machine regression (SVMR) models

One of the main challenges in the field of e-tongues is the implementation of models that can predict chemical parameters of importance in food quality control.

In this work, SVMR was used to predict acidity, density, and percentage of protein, lactose, and fat in milk. Sixty-five samples were used as the training set, and five samples (denoted V1, V2, V3, V4, and V5) were used as the external test set. The radial basis function, which could handle the non-linear relationships between the sensor signals and the target attributes, was chosen as the core function to predict acidity, density, and percentage of protein, lactose, and fat.

Although an electronic tongue has been shown to be able to perform classifications, the challenge for the reduced PE-tongue system is the implementation of regression models that can predict physicochemical parameters with adequate correlation values. For this purpose, SVMR has been applied to the 20-sensor array data.

Two data matrices have been built: the “X” matrix (predictors) constructed from the data recorded by the electronic tongue analyzing the milk samples and the “Y” matrix (responses) containing data of chemical parameters (acidity, density, protein, lactose, and fat) of the milk samples. Regression models were created using SVM regression (epsilon SVM, kernel type: radial basis function, C value: 1, cross-validation segment size: 15, and standard deviation weighting process in all cases).

The values obtained for the correlation coefficients and errors of calibration and prediction are shown in Table 4. In the case of acidity, protein content, lactose content, and density, the developed models achieved correlation values *R* ^2^ above 0.94 for both calibration and prediction, with low errors (RMSE) between 0.0239 and 0.9915. Lactose was the parameter with the lowest errors and highest correlation. In the case of fat, the correlation value only achieved 0.7789 for the prediction with a higher error of 0.6102.

**TABLE 4:** Results of the calibration and validation of SVMR for the 20-sensor e-tongue.

	Acidity	Density	%Proteins	%Lactose	%Fat
RMSE_c_	0.1642	0.4575	0.0275	0.0239	0.3819
R^2^_c_	0.9915	0.9684	0.9912	0.9901	0.9287
RMSE_p_	0.1988	0.6032	0.0341	0.0296	0.6102
R^2^_p_	0.9864	0.9452	0.9844	0.9844	0.7789

RMSE_c_: root mean square error of calibration; *R* ^2^_c_: correlation coefficient in calibration; RMSE_p_: root mean square error of prediction; *R* ^2^_p_: correlation coefficient in prediction.

The recognition capability of the five-sensor–based PE-tongue was tested by applying an SVM classification (SVMC) model. In this study, the model was trained with the data of 65 samples for the training set (corresponding to 13 types of milk samples depending on their fat content and nutritional content), calibrated against the true type, and used to predict the classification of the problem samples (five milk samples with different nutritional content) for the test set.

The values obtained for the correlation coefficients and errors of calibration and prediction are shown in Table 5.

**TABLE 5:** Results of the calibration and validation of SVMR for the five-sensor e-tongue.

	Acidity	Density	%Proteins	%Lactose	%Fat
RMSE_c_	0.3908	0.9299	0.6018	0.0373	0.8866
R^2^_c_	0.9311	0.869	0.9372	0.9763	0.6350
RMSE_p_	0.4522	1.0509	0.7005	0.0434	1.0286
R^2^_p_	0.9054	0.8316	0.9142	0.9666	0.3631

RMSE_c_: root mean square error of calibration; *R* ^2^_c_: correlation coefficient in calibration; RMSE_p_: root mean square error of prediction; *R* ^2^_p_: correlation coefficient in prediction.

As can be seen, in the case of acidity, protein content, lactose content, and density, the developed models reached values above 0.85 for both calibration and prediction, with low errors between 0.0373 and 1.0509, lactose being the parameter with lowest errors (0.0373 for the calibration and 0.0434 for the prediction) and the highest correlation (0.9763 for the calibration and 0.9666 for the prediction). However, in the case of fat, it is observed that a good correlation between the data provided by an electronic tongue and the physical–chemical data was not achieved.

Once the SVMR model was built, as a verification of its applicability, regression models were used to predict the physicochemical parameters (acidity, density, proteins, lactose, and fat) of a set of five external samples that were not included in the creation of the model. The results are shown in Table 6 vs. those obtained by traditional methods.

**TABLE 6:** Results of the prediction of chemical parameters in a set of five external samples.

	Sample 1	Sample 2	Sample 3	Sample 4	Sample 5
Acidity predicted	16.113	12.594	12.661	13.095	12.532
Acidity by traditional methods	15.94	12.36	12.45	12.95	12.45
% Relative error	1.07	1.86	1.69	1.16	0.64
Density predicted	1,038.831	1,031.995	1,031.97	1,031.463	1,031.452
Density by traditional methods	1,039	1,032.14	1,032.45	1,030.92	1,029.92
% Relative error	0.02	0.01	0.48	0.05	0.15
Proteins predicted	3.914	3.375	3.369	3.338	3.368
Proteins by traditional methods	3.89	3.34	3.35	3.29	3.33
% Relative error	0.51	1.2	0.6	1.52	1.2
Lactose predicted	5.464	4.976	4.98	4.937	0.531
Lactose by traditional methods	5.49	4.97	4.96	4.89	0.52
% Relative error	0.55	0.2	0.4	1.02	0.2
Fat predicted	0.41	1.665	1.716	2.572	2.31
Fat by traditional methods	0.35	1.58	1.65	3.12	3.58
% Relative error	40.20	5.06	4.24	17.63	35.47

The results obtained showed that the SVM regression model was able to predict the physicochemical factors with values that showed low relative errors with respect to the values obtained by traditional analysis techniques, the lowest errors being reached for lactose content and density. However, in the case of fat content, as expected given its correlation parameters, the values obtained by prediction show a high error, with the highest value being 2.11% in the case of skim milk. These results show that the model developed is unable to predict the fat content. In spite of the number of research works dedicated to the analysis of milk with potentiometric electronic tongues, the effect of the interaction between fats and the sensor membranes has not been discussed (Ciosek, 2016; Poghossian et al., 2019; Rodríguez-Mendez et al., 2016). A possible explanation is that when sensors are immersed in milk, fats participate in the formation of the double layer that creates the membrane potential. Once the double layer is formed, the excess of lipids does not contribute to the signal.

### Spoilage Monitoring

The capabilities of the simplified array were further evaluated by analyzing the capabilities to detect spoilage. For this purpose, tetrabrick packs were opened and stored at 5°C. Measurements were performed right after opening the packs and 5 days later.

**FIGURE 7:**
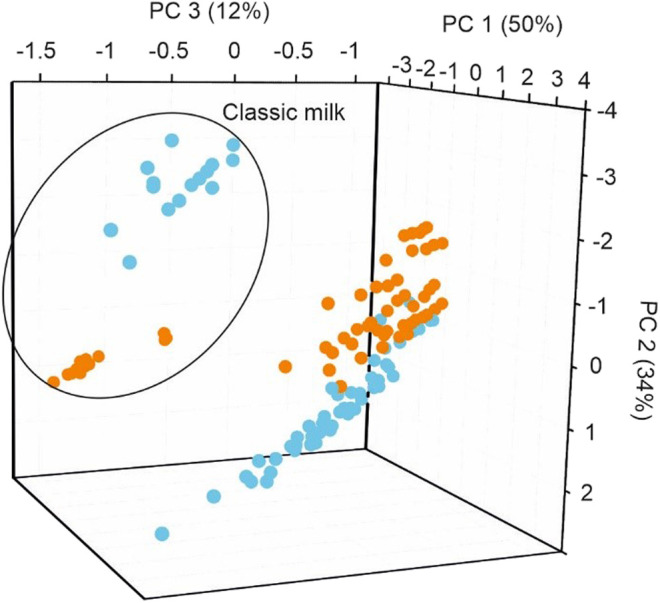
PCA score plot of the 13 milk samples analyzed using the PE-tongue: fresh sample (blue) and unsealed sample after 5°days (orange).

PCA was used as a first approach to evaluate the ability of the PE-tongue to detect spoilage occurring in unsealed milk. As shown in Figure 7, the classic milk sample appeared clearly separated from the rest of the milk samples, and the simplified PE-tongue could detect compositional changes occurring during storage. The reason why the classic milk samples (C-S, C-SS, and C-W) have a different aging behavior may be related to the fact that the rest of the milk samples have undergone various processes that can affect their composition. When examining the rest of the milk samples, a partial overlap between fresh and aged samples could be observed. This could be due to the fact that the degradation process does not occur at the same rate in all samples, as it can be affected by multiple factors such as milk composition and sample handling.

SVMC was used to classify between the two milk groups: fresh milk and aged milk. The classification results using the dataset showed that the sensor array achieved classification of the fresh and aged milk samples with an accuracy of 92.31% for the training set and 90.77% for the validation set. These results determine that the electronic tongue developed with five sensors was able to classify milk samples according to their state of preservation. Using SVMR, it has been demonstrated that the PE-tongue could be used to accurately predict physicochemical properties of the unsealed milk. After predicting the acidity of the aged samples, each of the samples showed an increase of approximately 1.2ºD in acidity. These results are consistent with the expected increase in milk acidity as a result of the increased microbiological activity in the unsealed samples.

## Conclusion

In this work, a simplified and portable electronic tongue (PE-tongue) was developed and used to predict chemical characteristics of milk samples. The system used only five potentiometric sensors that were selected from an extended array of 20 sensors using the genetic algorithm. PCA showed that the PE-tongue showed similar discrimination capabilities to the extended ET consisting of 20 sensors, but with a substantial decrease in the number of variables to be managed. The PE-tongue could be successfully used to classify milk with different nutritional characteristics and to predict acidity, density, %proteins, %lactose, and %fat, with low errors and high correlation coefficients. Potentiometric data acquired with the PE-tongue were successfully subjected to support vector machine (SVM) for classification of fresh and spoiled milk samples and to establish correlations with acidity of unsealed milk with excellent results.

## Data Availability

The raw data supporting the conclusions of this article will be made available by the authors, without undue reservation.
